# Useful In Vitro Techniques to Evaluate the Mucoadhesive Properties of Hyaluronic Acid-Based Ocular Delivery Systems

**DOI:** 10.3390/pharmaceutics10030110

**Published:** 2018-08-01

**Authors:** Angélica Graça, Lídia Maria Gonçalves, Sara Raposo, Helena Margarida Ribeiro, Joana Marto

**Affiliations:** 1 Laboratório Edol—Produtos Farmacêuticos, S.A., 2595-225 Linda-a-Velha, Portugal; angelicagraca@campus.ul.pt (A.G.); sraposo@edol.pt (S.R.); 2 Research Institute for Medicine (iMed.ULisboa), Faculty of Pharmacy, Universidade de Lisboa, 1649-003 Lisbon, Portugal; lgoncalves@ff.ulisboa.pt (L.M.G.); hribeiro@campus.ul.pt (H.M.R.)

**Keywords:** mucoadhesion, in vitro tests, hyaluronic acid, eye drops, dry eye disease, medical devices

## Abstract

Polymer-based eye drops are the most used drug delivery system to treat dry eye disease (DED). Therefore, the mucoadhesion between the polymer and the ocular mucin is crucial to ensure the efficacy of the treatment. In this context, the present study aimed to evaluate the potential use of in vitro methods to study the mucoadhesion of eye drop solutions and, specifically to evaluate the efficacy of two hyaluronic acid-based formulations (HA), HA 0.15% and 0.30% (*w*/*v*) to treat DED. Rheology methods and zeta potential determination were used to study the mucoadhesive properties of both eye drop solutions. All results indicated that interactions occurred between the mucin and the HA, being stronger with HA 0.30%, due to the physical entanglements and hydrogen bounding. In vitro tests on ARPE-19 cell line were performed using a 2D and a 3D dry eye model and the results have shown that pre-treated cells with HA showed a morphology more similar to the hydrated cells in both products, with a high survival rate. The in vitro techniques used in this study have been shown to be suitable to evaluate and predict mucoadhesive properties and the efficacy of the eye drops on relief or treatment of DED. The results obtained from these methods may help in inferring possible in vivo effects.

## 1. Introduction

Dry eye disease (DED), also known as keratoconjunctivitis sicca, is a pathology whose origin comes from several factors, resulting in symptoms of discomfort, visual disturbance, tear film instability with potential damage to the ocular surface, increased osmolality of the tear and inflammation. DED is a commonly reported clinical problem and the most frequently diagnosed disease in ophthalmology. Generalized inflammatory autoimmune diseases of the lacrimal gland are responsible for aqueous tear deficiency and excessive tear evaporation which causes the dry eye sensation [1,2,3]. Non-disease-related factors can also cause an alteration to the evaporation rate, including ambient conditions, hormonal regulation, blink rate, area of palpebral aperture, action of toxic topical agents such as preservatives and complication in the tear film compartments. Contact lenses can also disrupt the stability of the tear film, since they increase the evaporation rate, causing a rupture in the tear film about twice as fast as on the surface of the cornea [4,5,6].

The tear film is a three-layered structure with the purpose to provide protection and lubrication to the eye, reduces the risk of eye infection and keeps the surface of the eye smooth and clear. The lack of tear in the ocular surface may cause discomfort and dryness sensation [7,8,9].

There are several approaches to treat DED, until now most treatments do not treat the cause of the disease, but there are symptomatic treatments. Three forms of treatment are available: pharmacological, food-supplements and medical devices (MD). The first group, pharmacological treatment, is focused on treating inflammation and tear restoration, since DED may be a symptom of various illnesses, resulting in inflammation of the cornea and conjunctiva [10]. Food-supplements are concentrated sources of nutrients with physiological effect such as, for example, anti-inflammatory effect to attenuate symptoms of dry eye by treating chronic eye inflammation [11]. The third and final group, MD, is the most used form of treatment due to its simple administration and immediate relief. This includes MD such as tear supplements called artificial tears, which are artificial lubricants with hypotonic or isotonic buffers containing electrolytes, surfactants and many types of viscosity agents. Lubricant eye drops or artificial tears are the most common form to treat DED [1,2,3].

Lubricant eye drops differ in terms of composition (with polymers being the main excipient), viscosity, duration of action, presence and type of preservatives, osmolality and pH. The presence of polymers is crucial to improve mucoadhesive properties of artificial tears, in other words, the adhesion of a material to a mucous membrane or a mucus-covered surface, which is the case of the eye surface covered by mucin. Polymers used in artificial tears include hydroxypropyl methylcellulose (HPMC), carboxy methylcellulose (CMC), polyvinyl alcohol (PVA), Carbopol, polyvinylpyrrolidone (PVP), polyethylene glycol (PEG), dextran and hyaluronic acid (HA) [12,13,14].

There are several lubricant eye drops on the market with different types of polymers, each one with their own characteristics in terms of viscosity, retention time, mechanism and mucoadhesion properties. HA has been used with success in treating patients with severe DED. HA eye drops available on the market have a concentration ranging 0.10% and 0.30%, lower concentrations are used in less severe cases such as slight discomfort, and higher concentrations are more indicated for more severe cases, such as, risk of ocular damage [14]. The salt form of HA is sodium hyaluronate and its molecules can easily cover the corneal epithelium [14,15].

HA properties are similar to mucins, in terms of their viscoelasticity and biophysical properties. It has beneficial effects in providing a long-lasting hydration and retention time, obtaining a suitable lubrication of the ocular surface [14].

To understand the eye drop’s efficacy as a potential candidate to be used in the treatment of DED, the study of the mucoadhesion is of great importance. The most common and suitable methods to assess the mucoadhesive properties of a potential formulation candidate for ocular delivery is through in vitro techniques. Previous publications used simple methods which includes rheological techniques, particularly flow and oscillation methods and more sophisticated methods such as tensile strength measurements [16,17,18,19,20]. In vitro cell viability and morphology tests are also useful techniques to evaluate the efficacy of the product, studying the reaction of ocular cell lines, such as corneal epithelium HCE-T (Human Corneal Epithelial cells-Transformed) or ARPE-19 (Adult Retinal Pigment Epithelial cell line-19) cell lines, when exposed to the product [21].

Thus, the aim of this research work was to study the mucoadhesivity of two eye drop formulations containing HA, HA 0.15% and HA 0.30%, and to validate in vitro methods to be used in the future to study mucoadhesion properties in detail. A compilation of several rheological methods was performed, namely viscosity measurements, tackiness testing, and oscillation frequency sweep. The zeta potential (ZP) was also studied. As a complementary study of the product’s efficacy, a cell viability assay was performed using 2D and 3D culture cells models that mimic the conditions given by DED.

## 2. Material and Methods

### 2.1. Materials

High molecular weight (HMW) sodium hyaluronate (1.8–2.2 MDa), was a kind gift from Inquiaroma and N-hydroxymethylglycinate 50% (Suttocide) from Ashland (Covington, Kentucky, EUA, Brussels, Belgium). Potassium chloride, sodium chloride, sodium tetraborate and Ethylenediamine Tetraacetic Acid (EDTA) were purchased form VWR International (Carnaxide, Portugal), calcium chloride.6H_2_O was purchased from José Manuel Gomes Santos (Odivelas, Portugal), magnesium chloride.6H_2_O was purchased from Sigma-Aldrich Quimica (Sintra, Portugal) and boric acid was purchased from LaborSpirit (Loures, Portugal). In addition, for the mucoadhesive studies, it was used dried mucin from porcine stomach type II (Sigma-Aldrich, Saint Louis, MI, USA). Human retinal pigment epithelial cell lines ARPE-19 (ATCC^®^ CRL-2302™) were obtained from American Type Cell Culture collection (Manassas, VA, USA), and they were used for cell viability and dry eye assays. Cell culture medium and supplements were from Gibco (Thermo Fisher Scientific, Rochford, UK).

### 2.2. Methods

#### 2.2.1. Preparation and Characterization of HA 0.15% and HA 0.30%

To avoid eye irritation and provide ocular lubrication and comfort, there are some required specifications when formulating an eye drop solution. These specifications must obey the conditions existent in the ocular surface environment in terms of pH value, osmolality, electrolyte composition and sterility (Table 1). Formulation studies were performed and the excipients whose results were within specification were selected, namely potassium chloride, magnesium chloride hexahydrated, calcium chloride hexahydrated, sodium chloride for the electrolyte composition, boric acid and sodium tetraborat as buffering agents and Suttocide (N-hydroxymethylglycinate 50%) combined with EDTA as a preservative [22,23,24]. 

The preparation of both HA 0.15% and HA 0.30% (*w*/*v*) eye drop formulations starts with the introduction of highly purified water in an appropriate recipient and the addition of sodium hyaluronate, which is stirred until complete dispersion. Afterwards, the potassium chloride, magnesium chloride hexahydrated, calcium chloride hexahydrated, sodium chloride, boric acid, sodium tetraborate and EDTA were added and stirred until complete dissolution. The final step is the addition of the preservative Suttocide (N-hydroxymethylglycinate 50%), which is stirred, once again, until complete homogenization. The pH and the osmolality levels are adjusted with NaOH 40% or HCl 10%, and sodium chloride, respectively. The sterilization was carried out through a pre and sterile filtration process under an aseptic environment.

#### 2.2.2. Mucoadhesion Studies

The mucoadhesion was evaluated by viscosity, rheology and ZP measurements. The mucin used in this study was hydrated with water by gentle stirring until complete dissolution to yield a dispersion of 10% (*w*/*w*) at 20–25 °C.

##### Viscosity Measurements—Ostwald Viscometer

The viscosity properties of the eye drop solutions were determined at room temperature by using the Ostwald viscometer (Fisher Scientific, Pittsburgh, PA Hampton, NH, USA) using the following equation:

η_1_ = η_2_·ρ_1_t_1_/ρ_2_t_2_ (1)

where η_1_ and η_2_ are viscosity coefficients of the solution and water, ρ_1_ and ρ_2_ are the densities of the solution and water, and t_1_ and t_2_ are the flow times measured in the viscometer of the solution and water, respectively.

The viscosities of each individual component, HA 0.15%, HA 0.30% and mucin, were measure first in triplicate, a mean of the values of each component was made. To evaluate the effect of the interaction of the mucin with the solutions three samples were prepared: (1) 5% (*w*/*w*) mucin suspension; (2) Mucin suspension + HA 0.15% (*w*/*v*) solution (1:1) and (3) Mucin suspension + HA 0.30% (*w*/*v*) solution (1:1).

The mucoadhesion was expressed through the following equation:

Δ(%) = [η_muc+HA_ − (η_muc_ + η_HA_)]/(η_muc_ + η_HA_) × 100 (2)

where Δ(%) is the mucoadhesion index, η_muc_, η_HA_ and η_muc+HA_ is the mucin’s, the product’s and the solution containing mucin and product dynamic viscosity, respectively. For a mucoadhesive polymer, which is the case of HA, the η_muc+Ha_ is higher than (η_muc_ + η_HA_) due to the interactions occurring between the polymer and mucin. The mucoadhesive index is a measure of the mucoadhesive strength [18].

##### Rheology Measurements—Rotational Rheometer

The rheological characteristics of the formulations were examined at high shear rates using continuous shear techniques and in the viscoelastic region using oscillation techniques. These experiments were performed with a controlled stress Malvern Kinexus Rheometer (Malvern Instruments, Malvern, UK) using cone and plate geometry (truncated cone angle 4° and radius 40 mm). The frequency sweep method was performed between 0.1 Hz and 10 Hz, with a shear strain of 0.8%, at 25 °C, while the table of shear rate method was performed by increasing the shear rate from 0.1 s^−1^ to 100 s^−1^, at 25 °C. The shear stress was measured by this method and the apparent viscosity was calculated by dividing the shear stress by the shear rate.

An oscillatory amplitude sweep and frequency testing was performed using this equipment. The amplitude sweep conditions used were shear strain between 0.01% and 100% with the frequency of 1 Hz. It was concluded that the LVER (linear-viscoelastic region) was at shear strain of 0.25%. In the frequency testing the frequency range used was between 0.1–10 Hz with a shear strain of 0.25%. A time sweep test was also performed using this equipment with a shear strain of 0.25% and a frequency of 1 Hz during 30 min at 25 °C.

The adhesive strength was also measured using the same equipment and a plate and plate geometry (pull away assay or tackiness testing). It was used a toolkit with the conditions of 0.1 mm/s, 5 mm and 0.15 gap. The same protocol was performed using pig eyes obtained from a local slaughterhouse, instead of mucin. The eyes were attached to the probe and the adhesive force between the eye and samples were measured. While the probe is raised at a constant velocity it is measured the necessary force in which the sample dissociates from the probe, resulting in a force versus distance curve. The integral of that curve corresponding to the area under force-time curve represents the adhesive strength of the sample [22]. In this test the peak force which is a negative normal force resulted by the dissociated force between the probe and the sample can be attributed to tack and the area under the force-time curve represents the adhesive strength.

##### Zeta Potential (ZP)

The mucoadhesion interaction was also determined by measuring the ZP of the mixtures of mucin and each solution using a Zetasizer Nanoseries Nano Z (Malvern Instruments, Malvern, UK). A volume of 40 µL of all samples were diluted in 2 mL of filtered purified water and the cell was filled verifying for the existence of bubbles that could cause interference in the ZP measurements. All experiments were done in triplicate.

#### 2.2.3. In Vitro Cell-Based Assays

##### Cell Culture Condition

The ARPE-19 cell line (ATCC, CRL-2302™) was grown in DMEM/F12 culture medium (Gibco, Thermo Fisher Scientific, Rochford, UK) supplemented with 10 % (*w*/*v*) fetal bovine serum (FBS, Life Technologies. Inc., Thermo Fisher Scientific, Rochford, UK), penicillin (100 IU/mL) and streptomycin (100 μg/mL) in a humidified 95% O_2_, 5% CO_2_ environment at 37 °C. For the subculture, cells growing as monolayer were detached from the tissue flasks by treatment with 0.05% (*w*/*v*) trypsin/EDTA (Invitrogen, Thermo Fisher Scientific, Rochford, UK). The viability and cell count were monitored routinely using Trypan blue dye exclusion method.

##### Cell Viability of HA 0.15% and HA 0.30%

The cell viability was quantitatively evaluated in vitro using general cell viability endpoint MTT (3-(4,5-dimethyl-2-thiazolyl)-2,5-diphenyl-2H-tetrazolium bromide) reduction assay according the previously published procedure. MTT is a yellow and water-soluble tetrazolium dye that is converted by viable cells to a water-insoluble, purple formazan.

Cell viability was assessed after 24 h of incubation of ARPE-19 cell line with different concentrations of each sample. The negative control was the culture medium and positive control sodium dodecyl sulphate (SDS) at 1 mg/mL. After the time of exposition (24 h), the culture medium was replaced by medium containing 0.5 mg/mL MTT. The cells were further incubated for 3 h. In the plates containing reduced MTT, the media was removed, and the intracellular formazan crystals were solubilized and extracted with dimethylsulfoxide (DMSO). After 15 min at room temperature the absorbance was measured at 570 nm in a microplate reader (FLUOstar Omega, BMGLabtech, Ortenberg, Germany). The relative cell viability (%) compared to control cells was calculated by the following equations:

(3) $$\mathrm{Cell}\;\mathrm{Viability}\;\left(\%\right)\;\mathrm{for}\;\mathrm{the}\;\mathrm{MTT}\;\mathrm{assay}=\;\frac{\left[\mathrm{Absorvance}\;570\;\mathrm{nm}\right]\mathrm{sample}}{\left[\mathrm{Absorbance}\;570\;\mathrm{nm}\right]\mathrm{control}}\;\times 100$$

##### 2D Model—Evaluation of Cell Morphology and Cell Viability after Dehydration

The protective effect of the selected formulas against dehydration was evaluated using previously reported protocols, with modifications [18]. Specifically, cells were seeded in 24-well plates (5 × 10^4^ cells/well) and in DMEM/F12 until 70% confluence was reached. The medium was then replaced by the selected HA formulations diluted 1:5 in cell culture medium (HA 0.15%, HA 0.30% and a commercial formulation containing 0.30% of HA solutions). For the positive and negative controls, the medium was replaced with fresh medium not containing HA. Cells were incubated under cell culture conditions for 2 h. Cells treated with the HA samples and untreated cells (negative control, NC) were then dehydrated (about 20 min): the medium was removed and the plates without the lid were incubated at 37 °C until a stress response (morphological change) was evident in the NC. The positive control (PC, not treated with HA), was not dehydrated (cells were kept in the presence of the medium during all experiments).

Cell viability was evaluated using the endpoint resazurin reduction (7-hydroxy-3H-phenoxazin-3-one 10-oxide) (Alamar Blue) assay as described previously, cell viability (%) was calculated with respect to the PC (100% viability). Results were reported as means ± SD.

##### 3D Model—Dry Eye Model and Cell Viability

For the 3D dry eye assay, cells were cultured on filters following the protocol described by Dunn et al. with some modifications [23]. Briefly, the cells were seeded at a density of 1 × 10^5^ cells/cm^2^ on ThinCert™ cell culture inserts (Greiner, 3 μm, 12 wells, Stonehouse, UK). The culture medium was supplemented with l-ascorbic acid (50 µg/mL), β-glycerolphosphate (10 mM) and dexamethasone (10 nM) in order to enhance the barrier properties and facilitate expression of RPE-specific genes [23]. Fresh medium with supplements was changed twice a week.

The progress of epithelial barrier formation and polarization was followed by measuring Transepithelial Electrical Resistance (TEER) with a Millicell-ERS device (Merck Millipore, Darmstadt, Germany) and chopstick-style electrode. The combined resistance of the filter was subtracted from the values of filter-cultured ARPE-19 cells to calculate the resistance of the cell layer. The plateau in TEER was reached in two weeks and it remained essentially unchanged thereafter. The cells were used for experiments after culturing them for three weeks. In the permeability experiments the resistance was determined before and after the experiments.

After the three weeks ARPE-19 cells were placed under controlled environmental conditions to mimic dryness for two days (without lid, <40% relative humidity, 37 °C ± 5 °C temperature and 5% CO_2_). Cells were investigated for cell viability at 48 h after establishment of dry eye conditions, using the MTT reduction assay. Cell viability was assessed after 24 h of incubation of ARPE-19 cell line with 20 mg/mL concentration of each sample. The negative control was the culture medium and PC was SDS at 1 mg/mL.

##### Statistical Data Analysis

The data was expressed as mean and standard deviation (mean ± SD) of experiments. Tukey–Kramer multiple comparison test (GraphPad PRISM 5 32 software, La Jolla, CA, USA), was used to compare the significance of the difference between the groups, a *p* < 0.05 was accepted as significant.

## 3. Results

### 3.1. HA 0.15% and HA 0.30% (w/v) Formulation Development

Two eye drop solutions with different HA concentrations were developed, HA 0.15% and HA 0.30% (*w*/*v*). Both products are isotonic, with a limpid and clear aspect and a pH value between 7.0–7.6, which are similar to the lacrimal fluid to avoid eye irritation and provide ocular lubrication and comfort [24,25]. The lacrimal fluid electrolyte composition is mainly composed with Na^+^, K^+^, Cl^−^ and HCO^−^ and presents limited buffering capacity which is mainly due to the dissolved carbon dioxide and bicarbonate, thus the buffer selected must have low buffering capacity and significant antimicrobial activity. The antimicrobial activity of the buffer however itself is not sufficient to maintain the eye drop’s sterility, being the addition of and preservative a necessary step [24,25,26].

### 3.2. Mucoadhesive Studies

#### 3.2.1. Viscosity Measurements

A study using an Ostwald viscometer was also performed to evaluate the viscosity of the products and to study the interaction of HA 0.15% and HA.30% eye drop solution in the presence of mucin. The results obtained with an Ostwald viscometer presented in Table 2 shows HA 0.30% presents a much higher viscosity than HA 0.15%. An increase of viscosity was also observed in the presence of mucin, when compared with their individual viscosity. This increase is more evident for the HA 0.30%, with a viscosity value of 382.16 mPa·s and 53.20 mPa·s for the HA 0.15%. The mucoadhesive index was also calculated for the HA 0.15 + mucin and HA 0.30% + mucin solutions to evaluate the mucoadhesive strength gain due to the interactions between the polymer and the mucin (Table 2).

#### 3.2.2. Rheology Measurements

##### Oscillation Frequency Sweep

Before the oscillation frequency sweep, an amplitude sweep test was performed to define the fluid’s LVER, and the results showed that this region was at 25% shear strain. With this results the product’s structure can be further characterized using a frequency sweep proving more information about the effect of colloidal forces, interactions among particles or droplets [27].

**Figure 1:**
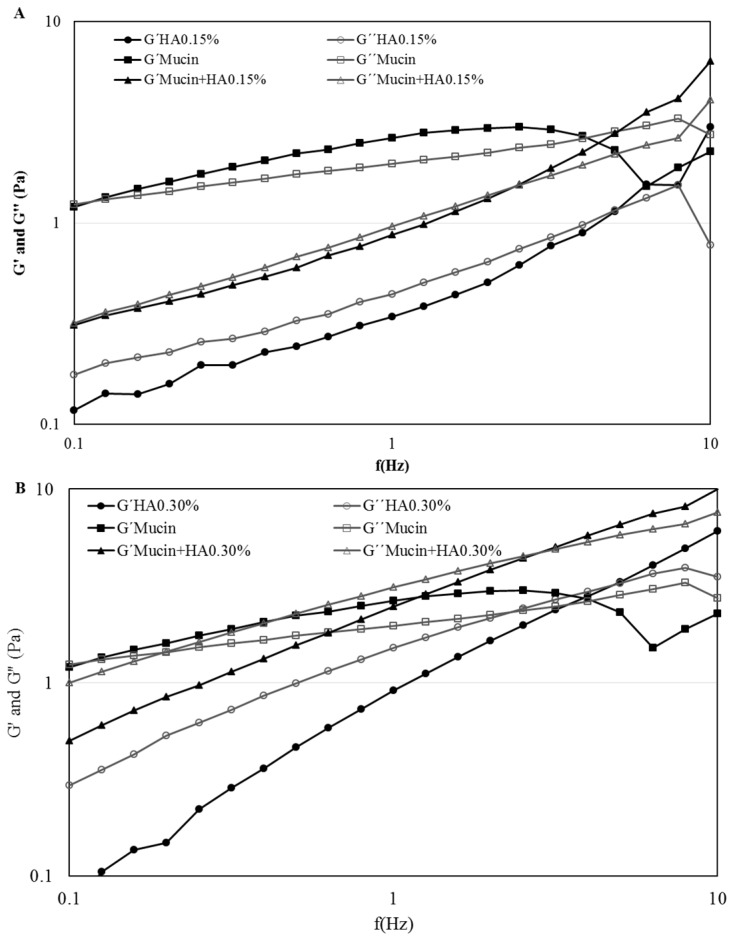
Frequency sweep with shear moduli as function of frequency of HA 0.15%, Mucin 5% and Mucin + HA 0.15% (**A**) and of HA 0.30%, Mucin 5% and Mucin + HA 0.30% (**B**) at room temperature.

Both Figure 1A,B represent the frequency behavior of the product, HA 0.15 % and HA 0.30% respectively, compared to system obtained with mucin. It is evident that with the mucin the elastic modulus G′ and the viscoelastic modulus G″ increased in both products. At lower frequencies both products exhibited fluid-like mechanism spectra with G″ modulus greater than G′, being both frequency dependent. As the frequency increases occurs a crossover at approximately 2–5 Hz, turning the G′ modules greater than the G″, indicating both products started to have a more elastic behavior.

##### Tackiness Testing

Tackiness in the context of material behavior is associated with stickiness and may result from adhesive forces between two materials in contact [22,28].

These parameters are measured, and the results of the prepared solutions are represented in Table 3. In this test, two commercial reference eye drop solutions (CR-Opticol^®^) with different concentrations of HA available on the market were used.

The results of the tack testing on the seven samples show that the mucin + HA 0.30% appears to be the tackiest of the seven samples analyzed with a peak normal force of −0.287 N, followed by the HA 0.30%, mucin 5%, CR 0.30%, mucin + HA 0.15%, HA 0.15% and CR 0.15% (Table 3). For the area under force-time curve the results did not show the same profile; the CR 0.30% appears to be the strongest of all samples (1.051 N·s) and the HA 0.15% the weakest (0.438 N·s).

A similar study was performed but instead of mucin were used three pig eyes. The results showed there are significant differences between HA 0.15% and HA 0.30% (*p* < 0.05), where the HA 0.30% appears to be tackiest with −0.134 N and the HA 0.15% with −0.078 N. The area under force-time curve also shows the same profile were HA 0.30% appears to be the strongest (Table 3).

##### Zeta Potential

**Figure 2:**
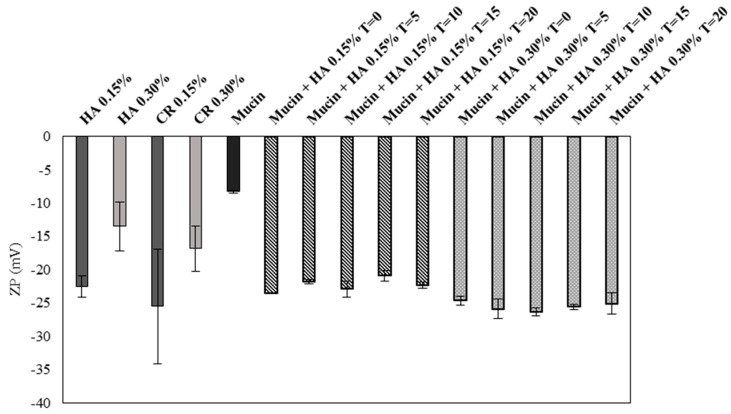
Determination of zeta potential for HA 0.15%, HA 0.30%, Mucin 5% and both products with mucin, values in absolute (Mean ± SD, n = 3).

ZP is related to the measurement of the surface charge that a specific material possesses or acquires when suspended in a fluid. This study demonstrated that the ZP values of HA 0.15% and HA 0.30% are similar to their market equivalent formulation, CR 0.15% and CR 0.30%, respectively. However, comparing the values of the two products their values are quite different being HA 0.15% ZP values much more negative that HA 0.30% values, which in absolute is higher (Figure 2). These negative values are in accordance with the anionic nature of the HA due to the presence of carboxylic groups. The mucin also presents negative charge due to the oligosaccharide chains which confer negative charge through carboxyl and sulphate groups. The obtained value is similar with the existing literature, which is approximately −10 mV [29,30]. When the mucin is added to both products an increase of the negative charge is observed, being the ZP value more negative in mucin 5% + HA 0.30% than with mucin 5% + HA 0.15%.

An overtime study was made to investigate if the interactions change overtime or if they maintain stable. The measurements of the samples were performed at 0, 5, 10, 15 and 20 min and it was concluded that the values do not suffer significant alteration overtime.

#### 3.2.3. In Vitro Cell-Based Assays

##### Cell Viability of HA 0.15% and HA 0.30%

**Figure 3:**
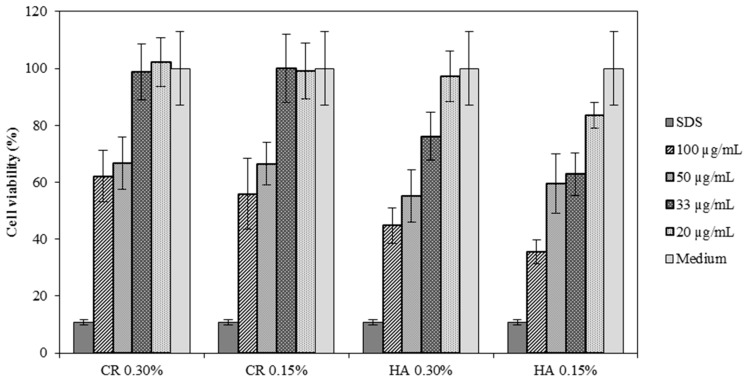
Results of cell viability on ARPE-19 cell lines testing CR 0.30%, CR 0.15%, HA 0.30% and HA 0.15% at various concentrations (mean ± SD, n = 8).

An initial test to evaluate the potential irritant of the HA formulations was performed to choose the most suitable dilution to be used on the next assays. Four dilutions were prepared 100 µg/mL (1:1), 50 µg/mL (1:2), 33 µg/mL (1:3) and 20 µg/mL (1:5). In Figure 3 it is evident that the 1:5 dilution presents a high rate of survival in all samples with cell viability above 80%. The 1:3 dilution although the CR samples demonstrated high survival rate (approximately 100%), the HA samples did not show the same results. For that reason, it was decided to use the 1:5 dilution on the next assays.

##### 2D Model—Evaluation of Cell Morphology and Cell Viability after Dehydration

In Table 4 it is shown optical microscope images of ARPE-19 cells stained with crystal violet and exposed to desiccation under no protective conditions (dry eye conditions, negative control), after being treated with HA 0.15%, HA 0.30% and CR 0.30% and of cells that were not exposed to dehydration (medium, PC). The respective cell viability determination of the samples is also present. In the dry eye images, it is evident the cells exhibited a disintegrated and dry membrane morphology and an increase of cell mortality. The cells treated with HA formulations did not show the same results, the typical morphology and high survival rate could still be observed. The results of cell viability confirmed the microscopic observation. The dehydration was responsible for almost 50% of mortality rate in the dry eye sample, while the cells pre-treated with HA formulations presented higher survival rates, 60–70%, confirming a protective effect displayed by the HA formulations.

##### 3D Model—Dry Eye Model Cell Viability

**Figure 4:**
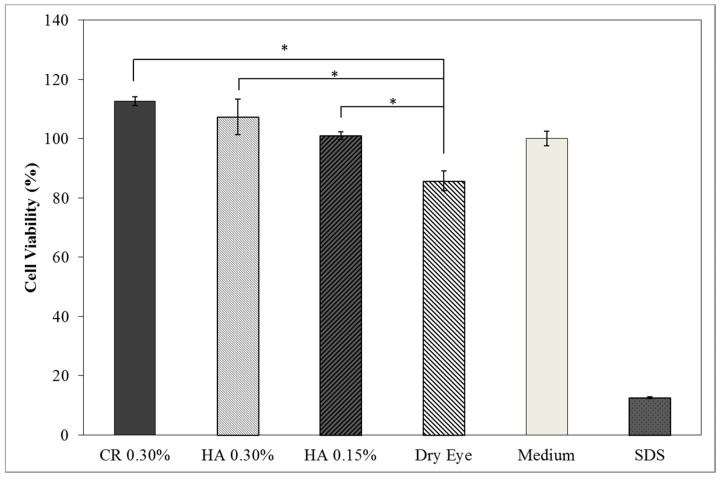
Cell viability of ARPE-19 cell lines exposed for 24 h to HA 0.15% and HA 0.30% formulations and commercial formulation (CR) containing 0.30% of hyaluronic acid (mean ± SD, n = 9). (* Significant statistical differences between both formulations *p* < 0.05).

The results of cell viability for the different tested samples are presented in Figure 4 showing a high cell viability profile of ARPE-19 cell line. When applied CR 0.30%, HA 0.30% and HA 0.15% cell viability increases when compared to the dry eye model. The survival rates were over 100% indicating that the formulations were non-toxic providing a suitable environment for cell proliferation.

## 4. Discussion

When designing an eye drop formulation, the mucoadhesive capacity is one of the most important aspects since it compromises the efficacy of the treatment. A product with low mucoadhesivity may not assure the necessary retention time to treat or relief symptoms of DED. A suitable in vitro method to evaluate this parameter is therefore an important aspect to understand the product characterization. Five studies were performed to understand the mucoadhesion properties of two eye drops, HA 0.15% and HA 0.30% (*w*/*v*) [31].

The first study was the determination of the viscosity thought an Ostwald viscometer. By measuring the viscosity, it is possible to evaluate the interaction of the product with the mucin, since a higher interaction with the mucin is related with higher viscosity. The results shown that HA 0.30% is much more viscous than HA 0.15% with 71.20 mPa·s and 6.83 mPa·s, respectively (Table 2). The suspension of mucin prepared for this research work (5%, *w*/*w*) presents viscosity since it is a high glycosylated protein with high MW.

To study the mucoadhesive properties of HA, samples of mucin with HA 0.15% and mucin with HA 0.30% were prepared and the results showed there are indeed interactions between mucin and HA, since the viscosity increased significantly when compared with the viscosity of HA 0.15% and HA 0.30% eye drop solution or mucin alone. The mucoadhesive index was also determined and it demonstrated both products have an increase superior to 50%. This increase suggests that a strong interaction between mucin and HA occurred, since HA 0.30% presents more concentration of HA, more interactions with the mucins were possible. This increase of viscosity given by the interactions between mucin and the polymer are possibly due to the formation of hydrogen bounding between the hydroxyl and carboxyl groups with mucin’s amino groups. The HA is a linear molecule and can easily interpenetrate a mucin random coil and that HMW polymers can increase the probability of interfacial interactions with mucin, creating a more stable connection. The same conclusions were assumed by Hassan and Gallo [32]. The adhesive capacity was not solely due to electrostatic bonding, which showed to be ambiguous, but also other types of bounding and interactions.

In non-ideal fluids the response of the polymer will depend on frequency with both shear moduli (G′ and G″) increasing with frequency. In both products, HA 0.15% and HA 0.30% eye drop solution, the G″ modulus is grater at low frequencies which indicates a fluid-like system (Figure 1A,B). With the addition of mucin this profile remains, but the values of both shear moduli increase. This means that it is necessary a greater amount of force or stress to deform the sample along the plane of the direction of the force, which indicates that some type of interaction has been established. According to Ludwig [12] the interaction between a mucoadhesive polymer and the mucin may occur by the following mechanisms: physical entanglements, Van der Walls bonds, electrostatic forces and hydrogen bonds. At low frequencies, the products and the system HA + mucin suffer a rearranging due to Brownian motion, physical entanglements are created and broken quickly compared to the rate of deformation, so they do not store elastic energy. At high frequencies the polymer/polymer + mucin system does not have time to rearrange causing the physical entanglements to persist longer than the oscillation frequency, constraining the polymer. The elastic energy is stored and the viscous dissipates, which is the reason at high frequencies the G′ moduli is higher than G″. Other explanation given by Cowman et al. [33] justifies the crossover due to the HA molecules relaxation time. When a solution is cyclically deformed, at slow rates the molecules are capable of keeping up with changes and behave in a viscous form. Then again rapid cyclic deformation does not allow the molecules to relax in shape, behaving more elastically, stretching and recoiling without flow. The crossover is the point of passage where the formulation stops presenting fluid-like characteristics and behaves more gel-like. The shear moduli present greater values in HA 0.30% in comparison with HA 0.15%, an indication that the strength of the formulation/mucin interaction increases with HA concentration [18,22].

The adhesive properties of polymer are highly influenced by the viscosity as well as the surface and interfacial tensions of the polymer and substrate. The results show that are significant differences (*p* < 0.05) between the normal peak force of HA 0.15% vs. HA 0.30%, HA 0.15% vs. Mucin + HA 0.15% and HA 0.30% vs. Mucin + HA 0.30% (Table 3). The first case makes sense since HA 0.30% has the double concentration of polymer, which makes it more viscous, as discussed before. The significant difference between HA 0.15% vs. Mucin + HA 0.15% and HA 0.30% vs. Mucin + HA 0.30% is an indication that the addition of mucin created an interference with the polymer which formed a more viscous system, concluding that a mucoadhesion occurred. The same conclusion was obtained with the pig eye, both normal peak force and area under force-time curve were higher with HA 0.30% than with HA 0.15%. Tensile strength assay on nasal, buccal and vaginal mucosa performed by Laffleur [34] using HA-based conjugates also showed similar adhesive bonding force between HA and the different mucosas, ranging between −0.1 and −0.2 N. According to Hägerström and Edsman, who performed a similar assay, the strengthening might arise from the entanglement of the polymer chains and the mucous glycoproteins, the formation of chemical bonds and/or from dehydration of the mucous layer [13]. However, the area under force-time curve values did not meet the same profile as the normal force, there were no significant difference between HA 0.15% vs. Mucin + HA 0.15% and HA 0.30% vs. Mucin + HA 0.30%. These results may be because the sample variability was higher in this measurement. Values obtained with mucin were higher than the ones with pig eye since the concentration of mucin is much higher in solution (5%, *w*/*w*) than the concentration existent in the eye surface.

The ZP results have shown that HA 0.15% and CR 0.15% ZP values are more negatively charged than HA 0.30% and CR 0.30% (Figure 2). As said before the negative charge of HA is due to the presence of carboxyl groups who dissociate at physiological pH [35]. By this reason, the higher the HA concentration the higher the negative charge. However, the HA in this product is at the form of sodium hyaluronate. Positively charged ions from the monovalent alkali metal series, such as Na^+^, act as counter ions on anionic structures such as HA, absorbing on the surface-dominating negative sites decreasing the absolute value of the ZP. These results are in accordance with Romero et al. and their work on silica with salts, which demonstrated that the ZP value of silica in absolute decreased with the addition of salts. The addition of salts reduces electrostatic repulsion which facilitates the formation of H bounds, and thus an increase in viscosity occurs [36].

With the addition of mucin both HA 0.15% and HA 0.30% ZP values increased in absolute, being the mucin + HA 0.30% a more negatively charged system. Mucin can be described as a double-globular protein region connected by highly glycosylated linkers, containing carboxylic and sialic acid, which confers the negative charge at physiologic pH. The overall net charge is negative, but there can also exist positively charge regions in the non-glycosylated globular region containing histidine, arginine and lysine residues. A study performed by Menchicchi et al. and other by Silva et al. concluded that the polymers such as chitosan present mucoadhesive properties due to the electrostatic interactions between positively charged polymer and negatively charged mucin [19,37]. However negatively charge polymers such as alginates, pectins and acrylic acid also show mucoadhesive properties, similar to the HA case. This means that the reason for mucoadhesion on polymers is not solely due to electrostatic interactions but also due to other types of interactions. Mucin forms a complex macromolecular network with available functional groups such as sialic acid, therefore it is possible to interact with the polymer by hydrogen bounding between sialic acid and the polymer’s carboxylate residue. It is also possible to form hydrophobic interactions with mucin’s amino acids and entanglement of the polymer. The changes in ZP value were more significant in HA 0.30% than HA 0.15% with the addition of mucin, because the first one presents double concentration of HA, more HA allows more interactions and thus an increase of viscosity [19].

The constant ZP values overtime are indicators that the system HA + mucin is stable. Stable interactions increase retention time, which means that the contact time in the corneal surface remains longer. Since the product in study is a MD, its action is of physical nature and not pharmacological, for that matter the longer the retention time the more efficient is the treatment [14,31].

Cell viability was performed using ARPE-19 cell line in a 2D and 3D in vitro model that mimicked DED conditions. By testing in these conditions, it is possible to study if HA 0.15% and HA 0.30% are promising candidates for the treatment of DED. A 2D in vitro assay was performed secondly to study the differences in the morphology and cell viability of dehydrated cells who received a pre-treatment with HA formulations, who were not submitted to the treatment (Table 4). It was also tested the commercial formulation CR 0.30% since this MD is used in severe cases of DED. The results have shown that the cells who were not treated with HA presented a disintegrated and dry membrane with a cell mortality rate close do 50% when compared to the non-dehydrated cells. The pre-treated cells showed a morphology more similar to the hydrated cells in both products with a high survival rate. In the 3D model the results show that the application of all three formulations obtained over 100% cell viability, meaning that the application of these MD provided a more suitable environment for cell proliferation. These results are very similar to the one performed by Salzillo et al. where it was performed a study of cellular response of primary porcine corneal epithelial cells when administrated different HA formulations [18]. They justified their results with the hypothesis that the protective effect displayed by the HA formulation on the cells is related to the polymer’s water retaining capacity. HA 0.30% presented higher cell viability values since more concentrated formulations retain more water, promoting higher hydration. These findings are important in the view of potential forms of treatment for DED, the higher the HA concentration the higher the efficacy, which concludes that HA 0.30% may be more indicated for more severe cases of DED.

When comparing the 2D with the 3D in vitro assay the results of cell viability performed in both methods were quite different. This is because the 2D cell culture model is more sensitive than the 3D model, this method only has a single layer which means that when submitted to dehydration all cells were exposed since all medium was removed. In the 3D model the dehydration is partial, the medium is removed in the insert, but it still exists in the well, simulating DED in vivo conditions.

## 5. Conclusions

The evaluation of mucoadhesion though in vitro methods allowed evaluation of HA 0.15% and HA 0.30% interactions with mucin, predicting their behavior in a biological environment.

The mucoadhesion between the HA and the mucin was tested and all results indicated that some kind of interaction occurred between the mucin and the HA, being stronger with HA 0.30%. Physical entanglements and hydrogen bounding are possible forms of interaction. The increase of viscosity when the mucin is added and the unchanged structure over time are indicators that the system HA + mucin is a stable interaction, which increases the retention time in the corneal surface, improving the efficacy of the treatment.

The cell viability test was performed with a 2D and 3D in vitro dry eye model. In the 2D model it was concluded that the cells pre-treated with HA preserved the cell’s morphology after the dehydration process and maintained a high survival rate. The 3D model demonstrated that the administration of HA 0.15% and HA 0.30% increased the cell viability over 100%. These values indicate that the application of HA favors cell proliferation by creating an optimum environment. The reason for that environment may be due to the hydration caused by the water retention capacity by the HA molecules.

The confirmation of strong mucoadhesivity and high cell viability are evidence that the products, HA 0.15% and HA 0.30%, are potential candidates for becoming suitable MD for DED treatment.

From these results the chosen in vitro methodology, viscosity, rheology and surface charge measurements, as well as cell viability assay, were demonstrated to be suitable to study mucoadhesivity in detail. The compilation of the results led to a deeper understanding of how the polymer can possibly interact with mucin, which infers possible effects on in vivo conditions.

## Figures and Tables

**Table 1:** Physical-chemical aspects of HA 0.15% and HA 0.30% (*w*/*v*) eye drop solutions.

Tests	Specifications	HA 0.15% Eye Drop Solutions	HA 0.3% Eye Drop Solutions
**Appearance**	Limpid, clear and odorless solution	Limpid, clear and odorless solution	Limpid, clear and odorless solution
**pH**	7.0–7.6 at 20–25 °C	7.16 (23.2 °C)	7.32 (20.8 °C)
**Osmolality**	280–320 mOsm/Kg	302 mOsm/Kg	304 mOsm/Kg
**Sterility (Ph. Eur. 2.6.1. Sterility)**	Absence of growth	Absence of growth	Absence of growth

**Table 2:** Viscosity values and mucoadhesive index for HA 0.15% (*w*/*v*) and HA 0.30% (*w*/*v*) in absence and presence of mucin 5% (*w*/*w*) (mean ± SD, n = 3).

Formulations	Viscosity (mPa·s)	Mucoadhesive Index (%)
**HA 0.15%**	6.8 ± 0.1	-
**HA 0.30%**	71.2 ± 4.1	-
**Mucin 5%**	25.0 ± 0.6	-
**Mucin 5% + HA 0.15%**	53.2 ± 1.1 *	298.07 ± 19.90 *
**Mucin 5% + HA 0.30%**	382.2 ± 0.4 *	67.44 ± 6.24 *

* Significant statistical differences between both formulations (*p* < 0.05).

**Table 3:** Normal force and area under force-time curve results for HA 0.15%, HA 0.30%, CR 0.15%, CR 0.30%, Mucin, Mucin + HA 0.15% and Mucin + HA 0.30% (Mean ± SD, n = 6).

Formulations	Peak Normal Force-Normal Force (N)	Area Under Force-Time Curve (N·s)
HA 0.15% *^1^	−0.178 ± 0.003 *	0.438 ± 0.058 *
HA 0.30% *^1^	−0.229 ± 0.013 *	0.775 ± 0.091 *
CR 0.15% *^1^	−0.168 ± 0.017	0.904 ± 0.069
CR 0.30% *^1^	−0.220 ± 0.007	1.051 ± 0.043
Mucin 5.0% *^1^	−0.228 ± 0.004 *	1.012 ± 0.065 *
Mucin 5% + HA 0.15% *^1^	−0.216 ± 0.019 *	0.573 ± 0.152 *
Mucin 5% + HA 0.30% *^1^	−0.287 ± 0.030 *	0.747 ± 0.066 *
Pig Eye + HA 0.15% *^2^	−0.078 ± 0.029 *	0.891 ± 0.060 *
Pig Eye + HA 0.30% *^2^	−0.134 ± 0.034 *	1.010 ± 0.059 *

*^1^ Mean ± SD, n = 6; *^2^ Mean ± SD, three different eyes, n = 3; * Significant statistical differences between both formulations *p* < 0.05.

**Table 4:** Optical microscope images of ARPE-19 after dehydration in no protective conditions (dry eye), after dehydration preceded by treatment with HA formulations and cells not submitted to dehydration (medium).

	Without Die Magnification 100×	Crystal Violet Magnification 200×	Crystal Violet Magnification 400×	Cell Viability (%)
**Dry Eye (negative control)**	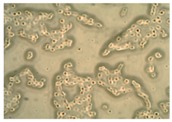	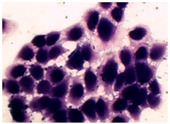	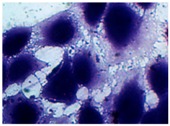	**50.7 ± 6.8**
**CR 0.30%**	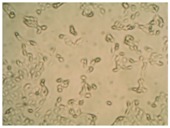	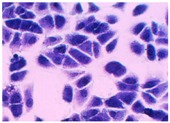	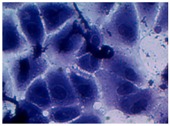	**73.4 ± 12.6**
**HA 0.15%**	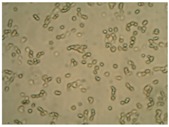	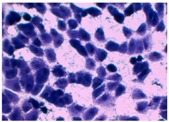	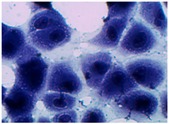	**62.7 ± 9.5**
**HA 0.30%**	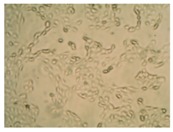	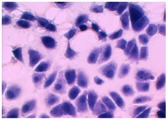	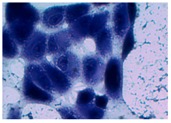	**72.5 ± 6.2**
**Medium (positive control)**	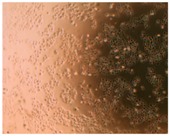	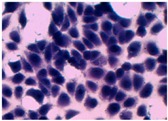	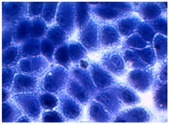	**100.0 ± 11.0**
